# Serum laminin γ2 monomer as a predictive biomarker for hepatocellular carcinoma in patients with chronic hepatitis B virus infection: a retrospective cohort study

**DOI:** 10.1038/s41598-024-77068-4

**Published:** 2024-10-25

**Authors:** Kouki Nio, Tetsuro Shimakami, Takeshi Terashima, Masahiro Yanagi, Tadashi Toyama, Naohiko Koshikawa, Masatoshi Nakagawa, Eisaku Yoshida, Toru Yoshimura, Motoharu Seiki, Masao Honda, Taro Yamashita

**Affiliations:** 1 Department of Gastroenterology, Kanazawa University Hospital, 13-1 Takara-Machi, Kanazawa, Ishikawa 920-8641 Japan; 2 Innovative Clinical Research Center, Kanazawa University Hospital, 13-1 Takara-Machi, Kanazawa, Ishikawa 920-8641 Japan; 3 Department of Life Science and Technology, Tokyo Institute of Technology, 4259 Nagatsuda-cho, Midori-Ku, Yokohama, Kanagawa 226-8501 Japan; 4 Diagnostics Division, Abbott Japan LLC, 357 Matsuhidai, Matsudo, Chiba 270-2214 Japan

**Keywords:** Cancer, Cancer screening, Tumour biomarkers

## Abstract

This retrospective study evaluated the use of laminin γ2 monomer (LG2m) as a predictive biomarker for hepatocellular carcinoma (HCC) in patients with chronic hepatitis B virus (HBV) infection. Serum LG2m levels were measured in two cohorts of patients: cohort 1 comprised 56 patients with chronic liver disease for assessing LG2m stability, whereas cohort 2 included 89 patients with chronic HBV infection who did not have HCC for evaluating the usefulness of LG2m measurement in HCC prediction. LG2m was highly stable in cryopreserved serum, and an increased LG2m level was significantly associated with a higher risk of HCC in chronically HBV-infected patients (P = 0.012). Multivariable Cox regression analysis revealed that high LG2m was an independent significant risk factor for HCC (hazard ratio, 7.16; 95% confidence interval, 1.31–39.2; P = 0.023). These findings suggest that LG2m may serve as a useful biomarker for the prediction of future HCC in patients with chronic HBV infection.

## Introduction

Hepatitis B virus (HBV) infection is a major cause of hepatocellular carcinoma (HCC), which is the most common primary liver malignancy and the third leading cause of cancer-related death worldwide^1^. Chronic HBV infection is associated with an increased risk of HCC, particularly in patients with cirrhosis^2^. Therefore, regular surveillance for HCC is recommended in patients with chronic HBV infection, particularly those with cirrhosis, in order to detect the tumor at an early stage when curative treatments are still possible. In addition to imaging studies, the use of biomarkers is also recommended for HCC surveillance in patients with chronic HBV infection. Several serum biomarkers, including alpha-fetoprotein (AFP), AFP-L3, and des-gamma-carboxy prothrombin (DCP), have been proposed as potential tools for the early detection of HCC. However, the sensitivity and specificity of these biomarkers remain suboptimal, and their clinical utility in HCC surveillance is still being evaluated. Thus, the development of useful biomarkers for HCC surveillance in chronically HBV-infected patients would be meaningful for the early detection of HCC and improved patient outcomes.

Laminin γ2 monomer (LG2m) protein has been identified as a potential biomarker for HCC surveillance because it is upregulated in HCC tissues^3^. Furthermore, in our previous multicenter prospective cohort study, we demonstrated the utility of serum LG2m measurement for HCC prediction in chronic hepatitis C patients who achieved sustained virological responses after treatment with direct-acting antivirals. However, the clinical utility of LG2m as a biomarker for HCC surveillance in patients with chronic HBV infection has not yet been fully evaluated. In this retrospective study, we investigated the utility of LG2m as a predictive biomarker for HCC in patients with chronic HBV infection.

## Results

### Stability of LG2m in cryopreserved serum

**Fig. 1:**
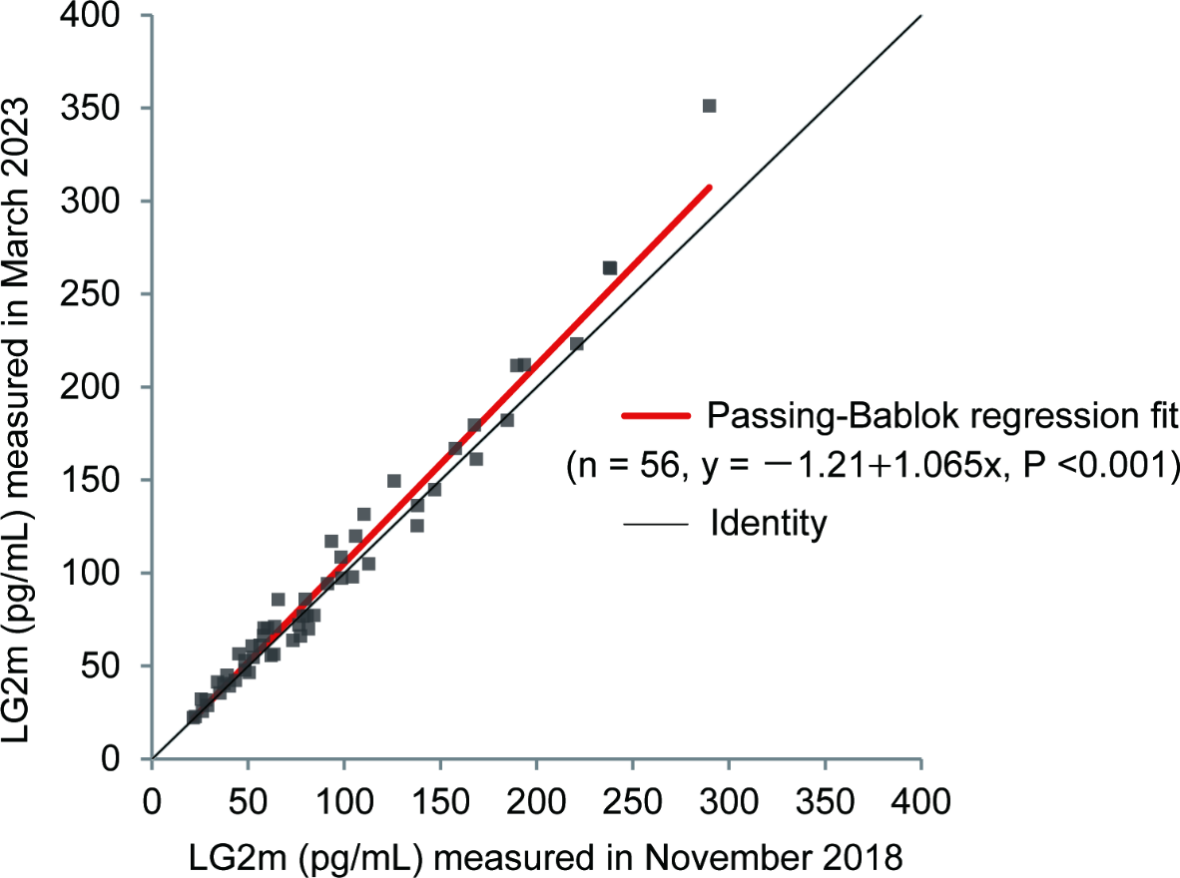
Approximation of LG2m in the same serum sample measured at two different times. Passing-Bablok regression fit comparing LG2m levels measured in 2018 and 2023 for cohort 1. The black line represents the line of identity (y = x).

Because this study used serum samples stored at − 20°C for more than 5 years, we first tested the reproducibility of the serum concentration of LG2m measured by using an Architect LG2m kit. We measured the LG2m concentration of thawed serum samples stored at − 20°C in 2018 and stored all samples again at − 20°C. We re-measured the LG2m concentration using the same serum samples in 2023 and analyzed the differences in the LG2m values measured at the different times (cohort 1). As shown in Fig. 1, LG2m was highly stable in serum that was repeatedly cryopreserved at − 20°C and thawed. These data demonstrate the utility of the LG2m measurement of patient-derived sera stocked at − 20°C for future use.

### Serum LG2m levels predict the incidence of future HCC in patients with chronic HBV infection

**Fig. 2:**
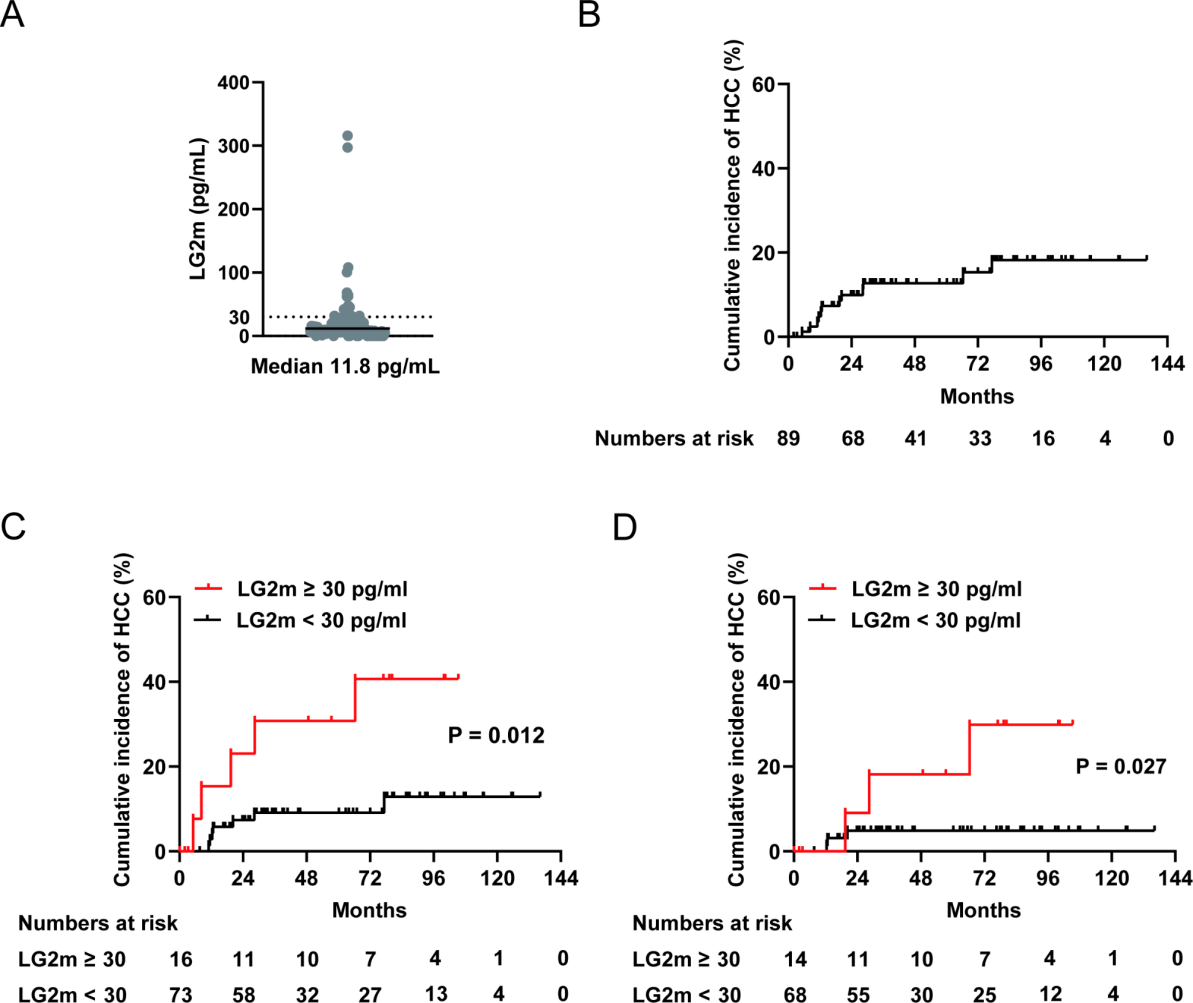
Cumulative HCC incidence in cohort 2. **A** Serum LG2m values for cohort 2. The cutoff value of 30 pg/mL is indicated by the dotted line. The median LG2m value is indicated by the black line. **B** Kaplan–Meier curve of HCC incidence in all enrolled patients. **C** Kaplan–Meier curves of cumulative HCC incidence in serum LG2m-high (red) and LG2m-low (black) HBV-infected patients. **D** Kaplan–Meier curves of cumulative HCC incidence in serum LG2m-high (red) and LG2m-low (black) HBV-infected patients without previous history of HCC. Number of patients at risk is shown at the bottom.

Patient characteristics of Cohort 2 are shown in Table 1, which included 7 patients (7.9%) with a history of HCC. The median LG2m level in the cohort of patients with chronic HBV infection (cohort 2) was 11.8 pg/mL (Fig. 2A). Of the 89 patients, 12 developed HCC during the follow-up period. The incidences of HCC were 4.9%, 9.9%, and 12.7% at 12, 24, and 36 months, respectively (Fig. 2B). In accordance with our previous study, in which we determined an LG2m cutoff value of 30 pg/mL for predicting HCC development, we defined LG2m-high as 30 pg/mL or higher. Of the 89 patients, 16 (18.0%) were categorized as LG2m-high. Elevated LG2m was associated with older age, hypoalbuminemia, a low platelet count, elevated fibrosis scores (APRI and FIB-4 index), and elevated tumor markers (AFP and DCP) but not with the previous history of HCC as shown in Table 2. Kaplan–Meier survival analysis demonstrated that an increased LG2m level was significantly associated with a higher risk of HCC (P = 0.012; Fig. 2C). Kaplan–Meier survival analysis of only patients without the previous history of HCC also showed that an elevated LG2m level was significantly associated with a higher risk of HCC (P = 0.027; Fig. 2D).

**Table 1:** Patient characteristics of Cohort 2.

	n = 89
Age, years (range)	54 (24–91)
Sex, % male	68.5
BMI, kg/m^2^	23.0
Diabetes, %	19.1
Alcohol use, % ≥ 20 g per day	19.8
Platelet count, × 10^4^/mm^3^ (range)	16.8 (1.7–63.0)
AST, IU/mL (range)	27 (13–433)
ALT, IU/mL (range)	28 (7–1067)
Albumin, g/dL	4.3 (2.4–5.1)
FIB-4 index (range)	1.83 (0.40–16.3)
APRI (range)	0.53 (0.11–7.50)
AFP, ng/mL (range)	3.0 (1.0–30.5)
DCP, mAU/mL (range)	22.5 (10.0–136.9)
Liver cirrhosis, n (%)	24 (27.0)
Previous history of HCC, n (%)	7 (7.9)
HBV-DNA, Log IU/mL (range)	4.9 (Undetectable- > 9.0)
HBsAg, COI (range) IU/mL (range)	> 2000 (< 0.1- > 2000) 2399.1 (102.7–11,617.5)
Use of nucleot(s)ide analogs, n (%)	64 (71.9)

**Table 2:** Clinical factors correlated with LG2m.

	LG2m-high	LG2m-low	P value
	n = 16	n = 73	
Age, years	61	53	0.050
Sex, % male	43.8	72.6	0.038
BMI, kg/m^2^	23.9	23.2	0.491
Diabetes, %	29.4	16.4	0.300
Alcohol use, % ≥ 20 g per day	11.8	21.4	0.506
Platelet count, × 10^4^/mm^3^	15.1	16.8	< 0.001
AST, IU/mL	45	25	0.378
ALT, IU/mL	34	28	0.800
Albumin, g/dL	3.9	4.4	< 0.001
FIB-4 index	4.54	1.53	< 0.001
APRI	1.40	0.45	0.009
AFP, ng/mL	8.6	2.8	< 0.001
DCP, mAU/mL	23.5	22.0	0.011
Previous history of HCC, %	12.5	6.8	0.605

To identify the potential risk factors for HCC development in these patients, we performed univariable Cox regression analysis using clinicopathological parameters. We found that diabetes, a low platelet count, a high Fibrosis-4 (FIB-4) index, a history of HCC, and LG2m were linked to a higher risk of HCC (Table 3). Although high LG2m was strongly confounded by a low platelet count and high FIB-4 index (Table 2), multivariable Cox regression analysis revealed that high LG2m, along with a history of HCC, was an independent significant risk factor for HCC (HR, 7.16; 95% CI, 1.31–39.2; P = 0.023) (Table 3). Taken together, the above data demonstrated the utility of serum LG2m measurement for the risk stratification of HCC in chronic hepatitis B patients.

**Table 3:** Univariable and multivariable Cox regression analyses of risk factors for HCC development.

Variables	Univariable		Multivariable	
	HR (95% CI)	P value	HR (95% CI)	P value
Age (+ 1 year)	1.03 (0.99–1.08)	0.201	1.00 (0.94–1.08)	0.919
Sex; male (vs. female)	0.61 (0.17–2.26)	0.459	0.51 (0.11–2.95)	0.509
BMI, kg/m^2^; ≥ 25 (vs. < 25)	2.17 (0.70–6.75)	0.179	-	-
Diabetes; yes (vs. no)	4.50 (1.44–14.0)	0.010	2.99 (0.83–10.7)	0.093
Alcohol, g per day; ≥ 20 (vs. < 20)	1.23 (0.35–4.80)	0.695	-	-
Platelet count, × 10^4^/mm^3^; ≥ 10 (vs. < 10)	0.20 (0.06–0.62)	0.005	0.40 (0.05–3.27)	0.389
AST, IU/mL; ≥ 40 (vs. < 40)	0.35 (0.08–1.61)	0.177	-	-
ALT, IU/mL; ≥ 35 (vs. < 35)	0.29 (0.06–1.34)	0.113	-	-
Albumin, g/dL; ≥ 4.0 (vs. 4.0)	0.77 (0.21–2.83)	0.689	-	-
FIB-4 index; ≥ 3.75 (vs. < 3.75)	3.00 (0.95–9.45)	0.061	0.63 (0.06–6.25)	0.696
APRI; ≥ 1 (vs. < 1)	1.67 (0.53–5.30)	0.384	-	-
AFP, ng/mL; ≥ 10 (vs. < 10)	0.62 (0.08–4.87)	0.557	-	-
DCP, mAU/mL; ≥ 30 (vs. < 30)	0.05 (0.00–1677)	0.533	-	-
LG2m, pg/mL; ≥ 30 (vs. < 30)	3.92 (1.24–12.4)	0.020	7.16 (1.31–39.2)	0.023
Previous history of HCC; yes (vs. no)	18.47 (5.88–58.0)	< 0.001	21.6 (4.67–99.7)	< 0.001

## Discussion

LG2m has been identified as a specific marker of cancer invasion that is highly expressed in several malignant tumor cells and in advanced invasive areas of various cancers, including HCC^4–7^. We recently demonstrated that LG2m is highly expressed in CD90-positive HCC cells, where conventional markers such as AFP and DCP are virtually absent. Furthermore, we reported that it is a useful biomarker for predicting patient prognosis and distant metastasis after curative surgery for HCC and future HCC development in hepatitis C virus (HCV)-infected patients after viral eradication^3^. However, the significance of LG2m as a predictive biomarker for HCC in patients with HBV infection, which is a major risk factor for HCC, similar to HCV, is unclear and was assessed in this study.

To ensure the reliability of the present retrospective study, because it was conducted using cryopreserved serum samples, we assessed the long-term stability of LG2m in samples stored at − 20°. Our results confirmed that LG2m is a stable protein, and the values of the same sample measured up to 5 years apart were highly consistent. We further measured the LG2m concentration in the cryopreserved sera obtained from patients with chronic HBV infection and examined its association with HCC development. In accordance with our previous study, we set the cutoff at 30 pg/mL in the present study. We found that 16 of 89 patients (18.0%) were classified as LG2m-high, which is slightly lower than the 30.6% in the previous HCV study^3^. The LG2m level increases with liver fibrosis and this result thus suggests a difference in the underlying liver fibrosis. In addition, the 1-, 2-, and 3-year rates of HCC incidence were higher than in the HCV study, possibly due to the enrollment of patients with a prior history of HCC, who were excluded from our previous prospective multicenter cohort study of chronic hepatitis C. Nevertheless, in the chronic hepatitis B cohort with this background, we found that LG2m was significantly associated with an increased risk of HCC, as revealed by Kaplan–Meier survival analysis and multivariable Cox regression analysis.

A predictive biomarker for HCC development refers to a biomarker that can identify individuals who are at high risk of developing HCC in the future. These biomarkers can help with the early detection and surveillance of HCC, enabling an earlier intervention and potentially better treatment outcomes. To stratify populations at risk for HCC development, we considered multiple risk factors for liver inflammation and fibrosis, including HBV, HCV, alcohol, and obesity. The significance of this study is that we have shown that LG2m is a predictive biomarker for HCC development in HBV-infected patients, building on the results of our previous HCV study.

The major limitation of this study is that it is a retrospective observational study of a small cohort that also includes patients with a history of HCC. We are presently conducting a prospective, multicenter study to investigate whether LG2m can be used as a biomarker to aid in the prediction of future HCC development. For this purpose, we are collecting serum samples from a substantial number of patients at risk of HCC development, including those with chronic HBV infection. We are eagerly awaiting the results of this validation study.

## Conclusion

Our findings provide insights into the potential clinical utility of LG2m as a predictive biomarker for HCC in patients with chronic HBV infection.

## Materials and methods

### Patients

This study enrolled two cohorts. Cohort 1 comprised 56 patients with chronic liver disease who had their serum cryopreserved for future analysis at Kanazawa University Hospital from January 2014 to December 2018. Cohort 2 comprised 89 patients with chronic HBV infection who were positive for hepatitis B surface antigen or hepatitis B e antigen but did not have HCC at the time of serum collection (includes patients with a history of HCC) and who had their serum cryopreserved for future analysis at Kanazawa University Hospital from January 2010 to December 2017. The study was conducted in accordance with the standards set by the Declaration of Helsinki and was approved by the institutional review board of the Graduate School of Medical Sciences, Kanazawa University.

### Measurement of serum LG2m

Serum LG2m level was measured by chemiluminescent immunoassay (CLIA) using a fully automated detection machine (Architect; Abbott Laboratories). LG2m-high was defined as 30 pg/mL or higher according to previous study^3^. 

### Statistical analysis

Passing–Bablok regression fit analysis was performed using Analyse-it (ver. 5.90; Analyse-it Software, Ltd.) to assess the stability of LG2m in cryopreserved serum. The time to HCC development was calculated as the interval between serum collection and the last follow-up visit or the date of HCC diagnosis. Cumulative HCC incidence was compared using log-rank tests, and Kaplan–Meier curves were generated using GraphPad Prism (ver. 9.3.0; GraphPad Software Inc.). Risk factors for HCC development were identified using Cox proportional hazards models and analyzed using SPSS software (ver. 28.0; IBM Japan, Ltd.). Unpaired *t*-test and Fisher’s exact test were performed using GraphPad Prism. A P value of less than 0.05 was considered statistically significant.

## Data Availability

The datasets used and/or analyzed during the current study available from the corresponding author on reasonable request.
